# Time-varying exposure to ambient air pollution and mortality among colon cancer patients in northern Thailand: a 15-year retrospective cohort study

**DOI:** 10.3389/fpubh.2026.1684020

**Published:** 2026-03-11

**Authors:** Taned Chitapanarux, Patrinee Traisathit, Pimwarat Srikummoon, Natthapat Thongsak, Nawapon Nakharutai, Salinee Thumronglaohapun, Titaporn Supasri, Phonpat Hemwan, Imjai Chitapanarux

**Affiliations:** 1 Division of Gastroenterology, Department of Internal Medicine, Faculty of Medicine, Chiang Mai University, Chiang Mai, Thailand; 2 Department of Statistics, Faculty of Science, Chiang Mai University, Chiang Mai, Thailand; 3 Data Science Research Center, Department of Statistics, Faculty of Science, Chiang Mai University, Chiang Mai, Thailand; 4 Atmospheric Research Unit of National Astronomical Research Institute of Thailand, Chiang Mai, Thailand; 5 Department of Geography, Faculty of Social Sciences, Chiang Mai University, Chiang Mai, Thailand; 6 Northern Thai Research Group of Therapeutic Radiology and Oncology (NTRG-TRO), Division of Radiation Oncology, Faculty of Medicine, Chiang Mai University, Chiang Mai, Thailand

**Keywords:** colon cancer, mortality rates, PM_10_, PM_2.5_, Thailand

## Abstract

**Background:**

Northern Thailand experiences severe annual air pollution (PM_2.5_ > 35 μg/m^3^), primarily due to agricultural burning. While ambient PM_2.5_ has been linked to gastrointestinal cancer deaths, its effect on colon cancer survival in high-exposure areas remains understudied.

**Methods:**

In this 15-year retrospective cohort study, we used data on 5,018 patients with colon cancer (2003–2018) obtained from the Chiang Mai Cancer Registry. Time-varying exposure to PM_2.5_, PM_10_, NO₂, SO₂, CO, and O₃ was evaluated by exploiting annually updated Copernicus Atmosphere Monitoring Service data and linking them to residential districts. Multivariable time-dependent Cox models adjusted for demographics, tumor characteristics, and treatment were used in the analysis.

**Results:**

Over 18,650 person-years of follow-up (PYFU), 72% of patients died (mortality rate: 19.4 per 100 PYFU). Exposure to PM_2.5_ ≥ 37.5 μg/m^3^ (a regionally relevant threshold) was independently linked to double the risk of all-cause mortality (adjusted hazard ratio (aHR) = 1.96, 95% confidence interval: 1.49–2.58, *p* < 0.001), as was having a low body mass index (aHR = 1.54) and being male (aHR = 1.23). Patients who died had significantly higher mean time-updated PM_2.5_ exposure (with consistent annual exposure differences) compared to the survivors (37.7 vs. 35.5 μg/m^3^; *p* < 0.001). No significant associations were found between colon cancer and PM_10_, O₃, NO₂, SO₂, or CO exposure. The PM_2.5_ effect size was larger than estimates from regions with lower exposure levels.

**Conclusion:**

Long-term exposure to ambient PM_2.5_ markedly increases the risk of death among colon cancer patients in northern Thailand. As a modifiable risk factor, PM_2.5_ mitigation urgently needs to be incorporated into environmental and survivorship care strategies in high-exposure areas.

## 1 Introduction

Ambient air pollution refers to the levels of pollution in outdoor air, which can vary significantly from one country to another. It includes harmful pollutants such as fine particulate matter ≤2.5 μm (PM_2.5_) or ≤10 μm (PM_10_), ozone (O₃), nitrogen dioxide (NO₂), sulfur dioxide (SO₂), carbon monoxide (CO), and volatile organic compounds (VOCs). These pollutants mainly originate from vehicle emissions, industrial activities, power plants, construction sites, and natural sources such as wildfires and dust storms. In northern Thailand, air pollution becomes especially severe during the dry season (January to April) due to agricultural burning. The annual average PM_2.5_ concentration often exceeds the World Health Organization’s guideline of 10 μg/m^3^ and reaches levels that pose a serious threat to public health (1, 2).

Ambient air pollution poses a significant public health threat due to its harmful impact on the respiratory and cardiovascular systems, resulting in various diseases and an increased risk of death (3, 4). Exposure to high levels of air pollution, particularly PM_2.5_, has been consistently linked to a greater likelihood of premature death (5). These tiny particles can reach deep into the lungs and enter the bloodstream, leading to chronic health issues such as heart disease, stroke, lung cancer, and respiratory conditions such as asthma and chronic obstructive pulmonary disease. Prolonged exposure to air pollution has been associated with a reduced life expectancy and an increased risk of early death, particularly for individuals residing in highly polluted regions (6, 7). Vulnerable populations, including children, the older adult, and individuals with pre-existing health conditions, are at even greater risk (8).

There is growing concern about the potential effects of ambient air pollution on the health of cancer patients and survivors. Their condition is often exacerbated due to the combined effects of cancer treatment (e.g., chemotherapy and radiotherapy) and air pollution, which can weaken cardiovascular and respiratory functions (9). In addition, these factors may lead to further health complications and also increase the risk of mortality (10).

Colon cancer is a leading gastrointestinal malignancy with high mortality both in Thailand and worldwide. A previous study in Thailand to explore colon cancer incidences and accumulated air pollution-related cancers between 2010 and 2016 found that increases in black carbon, organic carbon, and PM_2.5_ levels were associated with an increase in the risks of colon cancer (11). A recent study in China also found that long-term exposure to ambient air pollutants such as PM_2.5_, NO_2_, and SO_2_ was significantly associated with higher cancer and mortality risks among colorectal cancer patients (12, 13). Although long-term exposure to ambient PM_2.5_ has been associated with a higher risk of death from gastrointestinal cancers (14), evidence specifically on the connection between air pollution and colon cancer mortality remains scarce, especially for northern Thailand, an area which experiences the country’s highest seasonal PM_2.5_ pollution from biomass burning. This study aimed to examine the relationship between prolonged exposure to ambient air pollution and mortality risk among patients with and survivors of colon cancer, with particular attention to how this impact may evolve over time to fulfill a critical gap and provide region-specific evidence complementing broader studies of air pollution and cancer outcomes. This study’s setting was focused on northern Thailand due to this region’s severe seasonal PM_2.5_ pollution issues and the availability of a comprehensive cancer registry offering a large, high-quality dataset enabling robust time-varying exposure analyses.

## 2 Materials and methods

### 2.1 Study design and setting

This retrospective cohort study was conducted on data concerning patients diagnosed with colon cancer in upper northern Thailand (Chiang Mai, Chiang Rai, Lamphun, Phayao, Lampang, Nan, Phrae, and Mae Hong Son provinces), as well as provinces outside the northern region assessed collectively, to evaluate mortality and associated risk factors. We incorporated extended time-varying air pollution covariates (PM_2.5_, PM_10_, NO₂, SO₂, CO, and O₃) over an 18-year observation period (2003–2020) (15) and linked the environmental exposure data geographically to the district level within these provinces.

### 2.2 Participants

Eligible individuals included patients diagnosed with colon cancer (World Health Organization ICD-10 codes C18.0 to C18.9) between January 4, 1999, and December 28, 2018, as recorded in the Chiang Mai Cancer Registry. The participants in the study were followed up from the date of diagnosis until death, loss to follow-up, or censoring at the end of the follow-up period (May 15, 2024).

### 2.3 Variables and data sources

Patient demographic and clinical characteristics at diagnosis included sex, age, body mass index (BMI), smoking history, alcohol use, and cancer stage classified using the Surveillance, Epidemiology, and End Results (SEER) system (localized, regional, and metastatic). These data were obtained from the Chiang Mai Cancer Registry and the electronic database of Chiang Mai University Hospital, Chiang Mai, Thailand. Air pollution exposure data were obtained from the Copernicus Atmosphere Monitoring Service (CAMS), which provides hourly estimates of atmospheric pollutants at a spatial resolution of approximately 80 km through a combination of modeled outputs and observational data assimilation (T255 spectral format) (16). The CAMS Global Reanalysis (EAC4) has been validated and was considered to be highly reliable when applied in a recent Integrated Forecast System with 4D-Var data assimilation to integrate satellite observations (17). Hourly data from 2003 to 2020 were averaged to calculate the annual concentrations of each pollutant (18), which were then assigned to patients based on their district of residence recorded at the time of diagnosis.

### 2.4 Exposure assessment

Annual ambient air pollution exposure of each patient was estimated at the district level for each of the air pollutants while assuming that the patient lived at their recorded residence throughout the study period. Time-varying covariates were included in the analysis to account for changes in air pollution exposure throughout the study period.

### 2.5 Bias and study size

District-level pollution aggregation was applied for all participants to reduce misclassification bias in the exposure assessment. Selection bias was minimized through the comprehensive use of the cancer registry data that encompassed the entire regional population of colon cancer cases during the study period. All eligible patients were included in the analysis. No sample size calculation was necessary due to the census-like design.

### 2.6 Statistical analysis

Descriptive statistics were used to summarize the baseline characteristics. Categorical variables are reported as the frequency and percentage, while continuous variables are presented as the mean and standard deviation (SDs), or the median and interquartile range (IQR). The follow-up duration was calculated from the date of diagnosis to the earliest of the following events: all-cause death, last known follow-up, or the end of the observation period (December 31, 2020).

We hypothesized that the study variables with a high level of missingness could potentially confound the association with mortality in colon patients. Since complete-case analysis including these variables may lead to invalid findings, missing data in variables with over 30% missingness at baseline were imputed using multivariate techniques (19): linear regression for continuous variables (BMI with 49.6% missingness) and logistic regression for categorical variables (education and diabetes with 65.7 and 51.2% of missingness, respectively). Although smoking and alcohol histories had lower levels of data missingness (25.2 and 25.5%, respectively), the missing data for them were also imputed to enhance the accuracy of the analysis.

Overall and stratified mortality rates were expressed as the number of deaths per person-years of follow-up (PYFU). Confidence intervals (CIs) for mortality were estimated under the assumption of a Poisson distribution. Survival probabilities were calculated by using the Kaplan–Meier method, and differences between groups were assessed via log-rank tests. To address time-varying exposure, we fitted a time-dependent Cox proportional hazards model that included time-varying coefficients (20). Covariates included sex, age, cancer stage, smoking and alcohol-use histories, calendar year of enrollment, and annually updated air pollutant concentrations (PM_2.5_, PM_10_, NO₂, SO₂, CO, and O₃). The annually updated air pollutant concentrations were categorized based on official cut-off values provided by the Pollution Control Department, Ministry of Natural Resources and Environment, Thailand. The air pollution data were dichotomized according to the regionally relevant thresholds reported in previous studies as follows: PM_2.5_ (≥37.5 vs. <37.5 μg/m^3^) (21), PM_10_ (≥55 vs. <55 μg/m^3^), NO₂ (≥8.5 vs. <8.5 ppb), CO (≥410 vs. <410 ppb), and O₃ (≥36 vs. <36 ppb) (22, 23). Continuous exposure was categorized into quartiles or otherwise where relevant [e.g., BMI was classified as underweight (<18.5 kg/m^2^), normal weight (18.5–22.9 kg/m^2^), overweight (23–24.9 kg/m^2^), and obese (≥25 kg/m^2^)] (24).

Variables with *p*-values < 0.10 in the univariable models were considered for the multivariable analysis (conducted by using a backward elimination process). Highly correlated variables were excluded to reduce multicollinearity. All analyses were performed using STATA version 12 (StataCorp, College Station, TX) in accordance with the Strengthening the Reporting of Observational Studies in Epidemiology guidelines.

## 3 Results

Data on 5,018 colon cancer patients registered between January 2003 and December 2018 were used in the analysis. Among them, 2,236 (44.6%) were female and 2,782 (55.4%) were male, with a mean age of 59.4 years old, and most were over 50 years old (75.1%) and had a BMI of 22.0 kg/m^2^ (Table 1). The majority resided in northern Thailand (96.0%), particularly in Chiang Mai (54.2%). Primary education was the highest level for 51.7% of the population, and most were non-smokers (63.1%) and non-drinkers (61.7%). Few of them had diabetes (11.1%) or a family history of cancer (11.7%). Cancer was mainly found in the left colon (72.7%) and had mostly been classified as adenocarcinoma (88.3%). SEER classification identified the cancer incidences as local (42.6%), regional (28.7%), and metastatic (28.7%). Most patients had undergone surgery (82.4%) and chemotherapy (80.2%), with 48.4% also receiving radiotherapy. The mean follow-up period was 3.7 years, and the mortality rate was 71.9%.

**Table 1:** Baseline characteristics of the colon cancer participants (*N* = 5,018).

Variable	Category	*N* (%)
Sex	Female	2,236 (44.6%)
	Male	2,782 (55.4%)
Age (years old)	Mean ± SD	59.4 ± 13.7
	≤50	1,247 (24.9%)
	>50	3,771 (75.1%)
BMI (kg/m^2^)	Mean ± SD	22.0 ± 3.9
	<18.5	425 (16.8%)
	18.5–22.9	1,200 (47.5%)
	23.0–24.9	434 (17.2%)
	≥25.0	469 (18.6%)
Education	Unlettered	128 (7.4%)
	Primary school	889 (51.7%)
	Secondary school	352 (20.5%)
	Bachelor’s or higher	350 (20.4%)
Smoking history	No	2,369 (63.1%)
	Yes	1,383 (36.9%)
Alcohol-use history	No	2,309 (61.7%)
	Yes	1,431 (38.3%)
Diabetes	No	2,179 (88.9%)
	Yes	271 (11.1%)
Family history of cancer	No	3,186 (88.3%)
	Yes	421 (11.7%)
Tumor location	Transverse colon	728 (14.5%)
	Right-sided colon	644 (12.8%)
	Left-sided colon	3,646 (72.7%)
Pathology	Adenocarcinoma	4,430 (88.3%)
	Signet ring cell carcinoma	87 (1.7%)
	Neuroendocrine carcinoma	33 (0.7%)
	Other/Unspecified	468 (9.3%)
SEER cancer stage at diagnosis	Local	2,056 (42.6%)
	Regional	1,382 (28.7%)
	Metastatic	1,382 (28.7%)
Treatment received	Surgery	3,778 (82.4%)
	Chemotherapy	3,396 (80.2%)
	Radiotherapy	1,715 (48.4%)
Province of residence	Outside northern provinces	200 (4.0%)
	Northern provinces total	4,818 (96.0%)
	Chiang Mai	2,612 (54.2%)
	Chiang Rai	493 (10.2%)
	Lamphun	641 (13.3%)
	Phayao	311 (6.5%)
	Lampang	220 (4.6%)
	Nan	209 (4.3%)
	Phrae	144 (3.0%)
	Mae Hong Son	188 (3.9%)
Follow-up period (years)	Mean ± SD	3.7 ± 4.6
	Median (IQR)	2.0 (0.8–4.6)
Vital status	Deceased	3,610 (71.9%)

Table 2 reports the mortality rate per 100 PYFU by sex and age group. Over 18,650.5 PYFU, 3610 (72%) people died, with a mortality rate of 19.4 per 100 PYFU (95% confidence interval (CI): 18.7–20.0). By sex, 1,563 (70%) females died, with a mortality rate of 18.2 (95% CI: 17.3–19.1), while 2047 (74%) males died, with a mortality rate of 20.3 (95% CI: 19.5–21.2). By age, those 50 years or younger had a survival rate of 31% and a mortality rate of 18.0% (95% CI: 16.8–19.2), whereas those over 50 years had a survival rate of 27% and a mortality rate of 19.8% (95% CI: 19.1–20.6).

**Table 2:** Mortality rates by sex and age group.

Characteristic	Survived *n* (%)	Deceased *n* (%)	PYFU	Mortality rate*	95% CI
Overall	1,408 (28%)	3,610 (72%)	18,650.5	19.4	18.7–20.0
Sex					
Female	673 (30%)	1,563 (70%)	8,580.8	18.2	17.3–19.1
Male	735 (26%)	2,047 (74%)	10,069.7	20.3	19.5–21.2
Age (years)					
≤50	233 (31%)	859 (69%)	4,776.2	18.0	16.8–19.2
>50	1,020 (27%)	2,751 (73%)	13,874.3	19.8	19.1–20.6

*Reported as per 100 PYFU.

Table 3 summarizes the results of using the univariate and multivariate Cox proportional hazards models to analyze mortality risk factors. Males had a significantly higher mortality risk than females (hazard ratio (HR) = 1.09, 95% CI: 1.02–1.16, *p* = 0.01; adjusted HR (aHR) = 1.23, 95% CI: 1.02–1.48, *p* = 0.03). Age >50 years was associated with increased risk in univariate analysis (HR = 1.09, 95% CI: 1.01–1.18, *p* = 0.03), but not in the adjusted model (aHR = 1.17, 95% CI: 0.97–1.40, *p* = 0.11). Underweight patients (BMI < 18.5 kg/m^2^) had substantially higher mortality (HR = 1.25, 95% CI: 1.10–1.43, *p* = 0.001; aHR = 1.54, 95% CI: 1.25–1.90, *p* < 0.001). A higher education (bachelor’s degree or above) lowered the mortality risk (HR = 0.70, 95% CI: 0.55–0.90, *p* = 0.01; aHR = 0.64, 95% CI: 0.45–0.91, p = 0.01). Smoking history was associated with an increased risk in the univariate analysis (HR = 1.10, 95% CI: 1.02–1.19, *p* = 0.01) but not in the multivariate analysis (aHR = 0.84, 95% CI: 0.67–1.06, *p* = 0.15). Similarly, alcohol use showed a modest association in the univariate analysis (HR = 1.09, 95% CI: 1.01–1.18, *p* = 0.02) but not in the multivariate analysis (aHR = 1.00, 95% CI: 0.79–1.27, *p* = 0.97). Diabetes status was not significantly related to mortality in either model.

**Table 3:** Risk factors for mortality.

Characteristic	Deceased	Total	Univariable			Multivariable		
	(*n* = 3,610)	(*n* = 5,018)	HR	95% CI	*P*	aHR	95% CI	*P*
At colon cancer diagnosis								
Male	2,047	2,782	1.09	1.02–1.16	0.01	1.23	1.02–1.48	0.03
Age > 50 years old	2,751	3,771	1.09	1.01–1.18	0.03	1.17	0.97–1.40	0.11
BMI (kg/m^2^)								
<18.5	**317**	**425**	**1.25**	**1.10–1.43**	**0.001**	**1.54**	**1.25–1.90**	**<0.001**
18.5–22.9	760	1,200	Ref.	–	–	Ref.	–	–
23.0–24.9	247	434	0.85	0.73–0.98	0.02	0.91	0.72–1.15	0.44
≥25.0	254	469	0.74	0.64–0.86	< 0.001	0.90	0.72–1.12	0.35
Education level								
Primary school	675	889	0.92	0.74–1.14	0.44	0.81	0.60–1.11	0.20
Secondary school	247	352	0.83	0.66–1.06	0.13	0.75	0.53–1.05	0.09
Bachelor’s degree or higher	**219**	**350**	**0.70**	**0.55–0.90**	**0.01**	**0.64**	**0.45–0.91**	**0.01**
Unlettered	93	128	Ref.	–	–	Ref.	–	–
Behavioral/clinical factors								
Smoking history	1,038	1,383	1.10	1.02–1.19	0.01	0.84	0.67–1.06	0.15
Alcohol-use history	1,431	2,309	1.09	1.01–1.18	0.02	1.00	0.79–1.27	0.97
Diabetes	210	271	0.96	0.84–1.11	0.63	–	–	–
Tumor site								
Transverse colon	553	728	1.17	1.06–1.28	0.001	0.77	0.56–1.06	0.11
Right-side colon	436	644	0.95	0.86–1.05	0.32	**0.64**	**0.45–0.92**	**0.02**
Left-side colon	2,641	3,646	Ref.	–	–	Ref.	–	–
Pathological subtype								
Adenocarcinoma	3,109	4,430	Ref.	–	–	Ref.	–	–
Signet ring cell	**78**	**87**	**2.15**	**1.72–2.70**	**<0.001**	**3.89**	**1.96–7.71**	**<0.001**
Neuroendocrine carcinoma	25	33	1.34	0.90–1.98	0.15	1.16	0.47–2.89	0.75
Other/Unspecified	398	468	1.57	1.42–1.75	<0.001	0.74	0.50–1.09	0.13
Cancer stage								
Local	1,293	2,056	Ref.	–	–	Ref.	–	–
Regional	906	1,382	1.18	1.09–1.29	<0.001	1.15	0.95–1.40	0.16
Metastasis	**1,276**	**1,382**	**3.35**	**3.09–3.63**	**<0.001**	**3.35**	**2.74–4.09**	**<0.001**
Ambient air pollution exposure								
PM_2.5_ ≥ 37.5 μg/m^3^	**1,418**	**1,684**	**1.72**	**1.61–1.85**	**<0.001**	**1.96**	**1.49–2.58**	**<0.001**
PM_10_ ≥ 55 μg/m^3^	912	1,064	1.65	1.53–1.79	<0.001	0.89	0.63–1.26	0.53
NO_2_ ≥ 8.5 ppb	985	1,334	0.94	0.88–1.02	0.13	–	–	–
CO ≥ 410 ppb	1,006	1,181	1.69	1.57–1.82	<0.001	1.30	0.93–1.82	0.12
O_3_ ≥ 36 ppb	1,650	2,109	1.37	1.28–1.47	<0.001	0.92	0.77–1.11	0.39

Air pollution levels were estimated based on residential exposure between diagnosis and the last follow-up or death. Thresholds for PM_2.5_, PM_10_, NO₂, CO, and O₃ reflect regionally relevant concentrations based on air quality standards and epidemiological data. Bold values are results considered as statistically significant.

Tumor location also affected survival. Right-sided colon cancer was associated with a lower risk of death (aHR = 0.64, 95% CI: 0.45–0.92, *p* = 0.02) compared to left-sided colon cancer. Thus, the former can be used as the reference group since it demonstrated a higher mortality risk. Meanwhile, transverse colon cancers showed no significant effect (aHR = 0.77, 95% CI: 0.56–1.06, *p* = 0.11). Signet ring cell carcinoma was associated with a significantly higher mortality risk (HR = 2.15, 95% CI: 1.72–2.70; aHR = 3.89, 95% CI: 1.96–7.71, both *p* < 0.001), whereas unspecified cancers did not pose a significant mortality risk in the adjusted analysis. Regarding the SEER cancer staging, metastatic disease was strongly associated with higher mortality (HR = 3.35, 95% CI: 3.09–3.63; aHR = 3.35, 95% CI: 2.74–4.09, both *p* < 0.001), and higher mortality was seen in patients with regional disease in the univariate analysis (HR = 1.18, 95% CI: 1.09–1.29, *p* < 0.001) but not in the multivariate analysis (aHR = 1.15, 95% CI: 0.95–1.40, *p* = 0.16).

Pollution exposure also played a crucial role. PM_2.5_ ≥ 37.5 μg/m^3^ (a regionally relevant threshold reflecting the air quality context for northern Thailand) significantly increased the mortality risk (HR = 1.72, 95% CI: 1.61–1.85; aHR = 1.96, 95% CI: 1.49–2.58, both *p* < 0.001). In contrast, exposure to PM_10_ ≥ 55 μg/m^3^ was not associated with a higher mortality risk after adjustment (aHR = 0.89, 95% CI: 0.63–1.26, *p* = 0.53). Elevated CO levels (≥410 ppb) were significant in the univariate analysis (HR = 1.69, 95% CI: 1.57–1.82, *p* < 0.001) but not after adjustment (aHR = 1.30, 95% CI: 0.93–1.82, *p* = 0.12). Similarly, ozone (O₃) exposure (≥36 ppb) did not significantly affect mortality in the multivariate model (aHR = 0.92, 95% CI: 0.77–1.11, *p* = 0.39).

Of the 5,018 colon cancer patients included in the study, data on ambient air pollution were available for 4,514 of them only. Among these, those who died (*n* = 3,246) had significantly higher time-updated exposure to both PM_2.5_ and PM_10_ compared to the survivors (*n* = 1,268) (Table 4). Specifically, the mean PM_2.5_ concentration exposure for the deceased (37.7 ± 8.4 μg/m^3^) was notably higher than that for the survivors (35.5 ± 7.0 μg/m^3^, *p* < 0.001). Similarly, the mean PM_10_ exposure was 52.4 ± 11.6 μg/m^3^ for the deceased group compared to 49.5 ± 9.6 μg/m^3^ for the survivors (*p* < 0.001). The median results also supported these findings.

**Table 4:** Comparison of time-updated PM_2.5_ and PM_10_ concentrations from diagnosis to last follow-up or death.

Summary statistics	Total (*n* = 4,514)	Survived (*n* = 1,268)	Deceased (*n* = 3,246)	*p*-value
Number of pollutant records* (PM_2.5_ and PM_10_)	19,732	6,860	12,872	–
Time-updated PM_2.5_ (μg/m^3^)				
Mean (SD)	36.9 (8.0)	35.5 (7.0)	37.7 (8.4)	<0.001
Median (IQR)	36.6 (32.5–39.5)	35.2 (31.0–38.7)	37.0 (33.1–40.4)	<0.001
Time-updated PM_10_ (μg/m^3^)				
Mean (SD)	51.4 (11.0)	49.5 (9.6)	52.4 (11.6)	<0.001
Median (IQR)	50.9 (45.3–55.0)	49.0 (43.2–53.9)	51.7 (46.2–56.1)	<0.001

*PM concentrations were modeled as annually updated averages from diagnosis until death or last follow-up. *p*-values were obtained from independent-sample *t*-tests or Mann–Whitney *U* tests where appropriate. Units: μg/m^3^ values are presented as the median (IQR) or the mean ± SD where appropriate.

**Figure 1:**
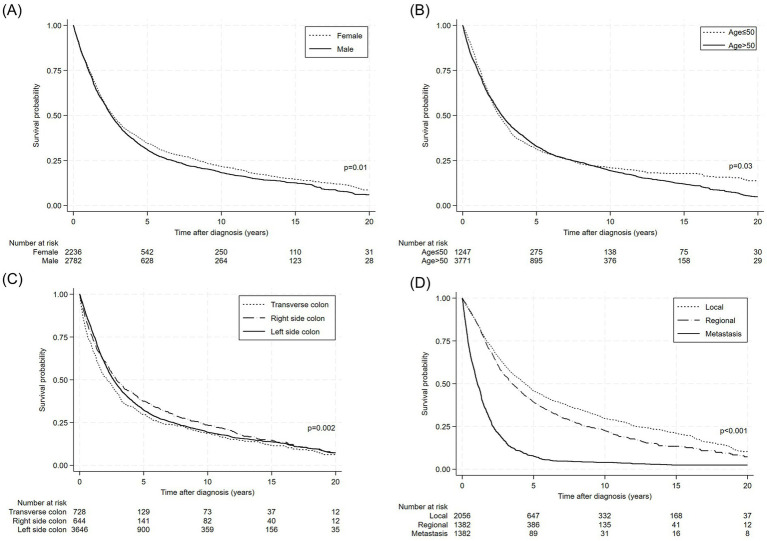
Kaplan–Meier survival curves for the colon cancer patients stratified by **(A)** sex, **(B)** age group at diagnosis, **(C)** tumor site, and **(D)** cancer stage.

Figure 1 illustrates Kaplan–Meier survival curves for the survival probabilities of colon cancer patients categorized by sex, age at diagnosis, tumor site, and SEER cancer stage. Overall, the curves emphasize prognostic diversity within the study population. For instance, patients diagnosed at a younger age or with an earlier-stage cancer generally had a higher probability of survival over time (the curves declined more gradually). Tumor site also affected prognosis, with different types of colon cancer showing different patterns. In addition, male and female patients had different survival outcomes, indicating possible biological or care-related disparities.

**Figure 2:**
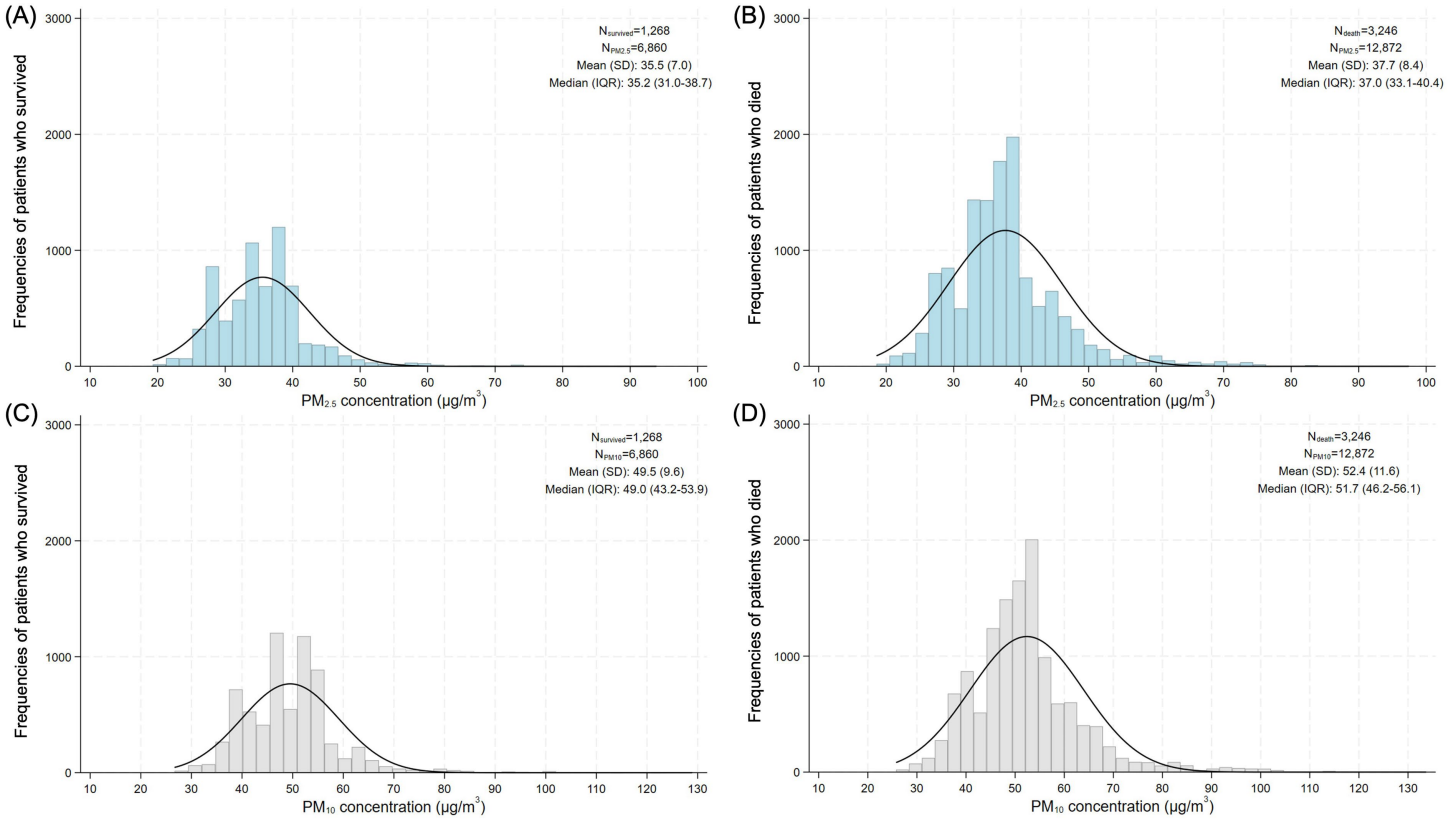
Average PM_2.5_ exposure concentrations according to colon cancer survival **(A)** versus deceased **(B)**. Average PM_10_ exposure concentrations according to colon cancer survival **(C)** and deceased **(D)**. Air pollutant exposure was estimated from diagnosis to the last follow-up or death.

Figure 2 shows the distribution of the long-term exposure of the colon cancer patients to PM_2.5_ and PM_10_ divided by survival status. Figures 2A,B illustrate how the annually averaged PM_2.5_ levels varied between the survivors and the deceased, respectively, while panels Figures 2C,D show the same comparison for PM_10_. Since exposure was modeled from diagnosis to last follow-up or death, the distributions reflect the patients’ cumulative environmental burden during their disease course. This perspective enables the evaluation of whether higher pollution exposure levels were more common among those who died, thereby providing evidence for a potential link between air pollution and mortality risk.

**Figure 3:**
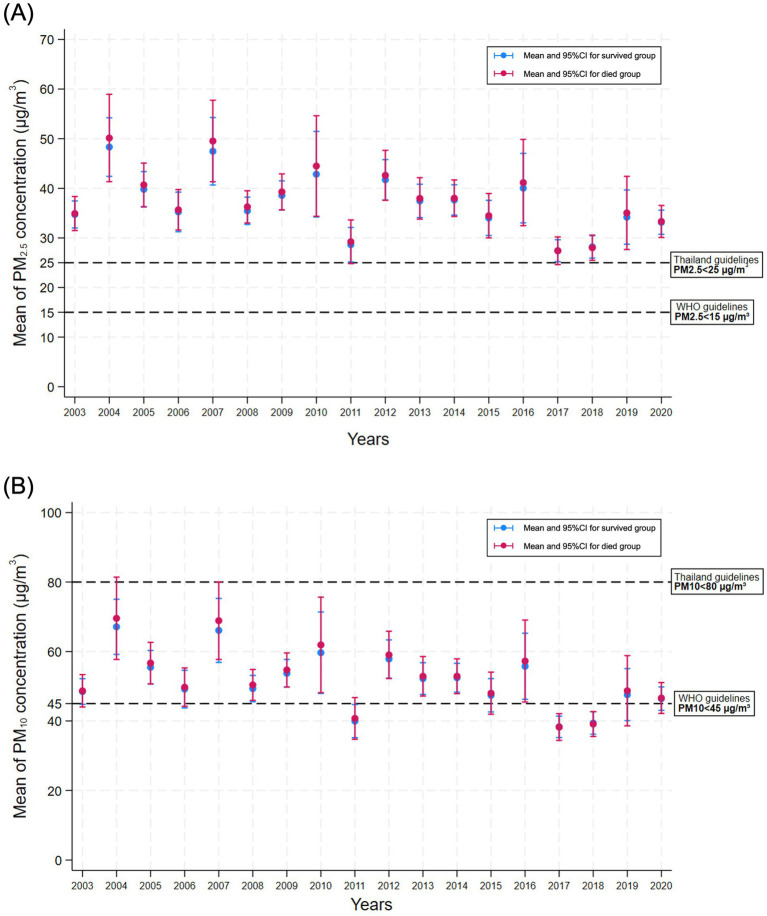
Line plots illustrating the annual mean concentrations of **(A)** PM_2.5_ and **(B)** PM_10_ from the year of colon cancer diagnosis to the last follow-up or death. Data are stratified by survival status (survived vs. deceased). Exposure estimates are based on the patients’ residential location and account for time-varying air quality measured throughout the follow-up period.

Figure 3 illustrates the annual trends in PM_2.5_ and PM_10_ exposure from diagnosis to the last follow-up or death among the patients with colon cancer stratified by survival status. Patients who died consistently experienced higher PM_2.5_ levels, highlighting the potential link between sustained air pollution exposure and increased mortality risk.

## 4 Discussion

This 15-year retrospective cohort study offers strong evidence that long-term exposure to ambient PM_2.5_ is an independent and substantial predictor of all-cause mortality for colon cancer patients in northern Thailand. Patients exposed to PM_2.5_ ≥ 37.5 μg/m^3^ experienced nearly double the risk of death (aHR = 1.96, 95% CI: 1.49–2.58), an effect exceeding that of being male (aHR = 1.23) or underweight (aHR = 1.54), and second only to metastatic disease (aHR = 3.35) and signet ring carcinoma (aHR = 3.89). These findings, underscoring the growing importance of assessing the effect of air pollution exposure on cancer survival, expand on previous research. Compared to a US cohort study that reported a 17% increase in gastrointestinal cancer mortality for every 10 μg/m^3^ rise in PM_2.5_ (25), and a recent meta-analysis that estimated a pooled colorectal cancer mortality odds ratio of 1.21 (26), our study shows a stronger association between high ambient PM_2.5_ exposure and death from colon cancer. This larger effect may be due to region-specific risks in northern Thailand, where annual PM_2.5_ levels consistently exceed 35 μg/m^3^ (much higher than in western Thailand). This is mainly caused by seasonal agricultural burning that results in air pollution that is high in ultrafine particles, heavy metals, and VOCs. In addition, this increases the mortality risk of cancer patients who already have health challenges posed by cancer treatment regimens and limited healthcare access.

Our finding indicating that long-term PM_2.5_ exposure induces a higher risk of mortality among colon cancer patients is consistent with that in a previous study in China (13), which found that each unit increase in PM_2.5_ is associated with a higher rate of all-cause mortality. However, the findings according to some pollutants were inconsistent.

The lack of association between the mortality risk of the study participants with other air pollutants such as PM_10_ (*p* = 0.53), O₃ (*p* = 0.39), CO (*p* = 0.12), or NO₂ (*p* = 0.13 in the univariable analysis) reinforces the particular danger of PM_2.5_ exposure. Mechanistically, PM_2.5_’s small diameter allows it to bypass mucociliary defenses, reach the alveolar space, and translocate into the systemic circulation. Once internalized, it can facilitate DNA damage, chronic inflammation, endothelial dysfunction, and epigenetic alterations, all of which have been implicated in cancer progression and mortality (27–30). In contrast, coarser particles and gaseous pollutants may exert more localized effects or be less capable of inducing systemic pathophysiological responses in vulnerable oncological populations.

Our findings are consistent with evidence from Hong Kong (7) and Taiwan (14), reinforcing regional relevance and highlighting the disproportionate burden of gastrointestinal cancer mortality in high-pollution settings across LMICs and East Asia. While acknowledging the methodological constraints of our study, the findings provide important insights. We have provided region-specific epidemiological evidence that enhances the global understanding of how air pollution exposure, particularly ambient PM_2.5_, affects colon cancer outcomes. In high-exposure, resource-limited areas such as northern Thailand, PM_2.5_ stands out as a modifiable and biologically plausible risk factor with significant prognostic consequences. These results highlight the importance of incorporating environmental risk assessments into cancer survivorship care, particularly in Southeast Asia, where seasonal biomass burning poses a persistent threat to health. To better assess the mortality risk, future research should use higher-resolution exposure models (e.g., land-use regression, satellite calibration, etc.), incorporate molecular tumor profiling, and systematically gather socioeconomic and behavioral data. Meanwhile, educating patients, improving household air quality, and regionally tailoring air pollution mitigation policies could help reduce air pollution exposure and improve the outcomes for vulnerable populations.

These findings provide important insights while acknowledging methodological constraints. A major strength is the use of satellite-derived air pollution data from the Copernicus Atmosphere Monitoring Service, which improved spatial accuracy in exposure estimation across Northern Thailand. Time-updated pollutant exposures aligned with each patient’s follow-up captured dynamic environmental burdens more precisely than static models. This dynamic approach more accurately captures the cumulative environmental burden and enables stronger causal inferences than static baseline models. The clear exposure gradient observed between survivors and non-survivors (both in overall metrics and yearly trends) underscores the temporal and dose-dependent nature of the association, especially in a region such as northern Thailand, where air pollution levels vary greatly due to seasonal biomass burning. The large, population-based cohort from the Chiang Mai Cancer Registry minimized selection bias and ensured statistical power. However, exposure assessment relied on district-level residential data at diagnosis, assuming stability throughout follow-up, which may cause nondifferential misclassification if patients relocated. Future work should apply finer-resolution models (e.g., land-use regression, satellite–ground calibration) and longitudinal residential histories to reduce bias.

Several limitations of the study must be acknowledged. District-level exposure estimates (~80 km resolution) may lead to nondifferential misclassification, particularly given the assumption of the patients not changing residence. This likely weakens the true effect estimates. Next, extensive imputation of missing data for BMI and education level may have reduced the accuracy of adjusting for socioeconomic status. Behavioral covariates such as smoking and alcohol use lost significance after adjustment, possibly due to residual confounding or measurement errors. Sensitivity analysis to compare the results of complete-case analysis using the data with and without imputation should be conducted to investigate this issue. Although diabetes status was included in the registry, other comorbidities such as cardiovascular and respiratory diseases were not consistently recorded. Thus, residual confounding from unmeasured comorbidities might have influenced the observed associations. Furthermore, other potentially confounding factors that might have influenced the association, such as diet, exercise, occupational exposure, family income, and local environmental contamination, were not included in this study due to the data not being available. These factors should be included to investigate potential confounding effects in a future study. Finally, although our findings support a direct biological effect of PM_2.5_ on colon cancer mortality, high pollution levels may also reflect broader contextual disadvantages, including poor environmental conditions, socioeconomic challenges, and limited healthcare access, which together could exacerbate survival disparities in Southeast Asia.

## 5 Conclusion

This study established that a high ambient PM_2.5_ level has a significant effect colon cancer survival in northern Thailand, thereby underscoring the urgent need for environmental health strategies in oncological care for regions plagued by to air pollution. Beyond clinical outcomes, our findings carry important public health implications. Cancer care in Southeast Asia should incorporate environmental risk assessments and patient education, while regional policy reforms aimed at reducing biomass burning and ambient pollution are urgently needed to reduce exposure and improve survivorship.

## Data Availability

The datasets generated and/or analyzed during the current study are available from the corresponding author on reasonable request. Requests to access the datasets should be directed to Imjai Chitapanarux, imjai.chitapanarux@cmu.ac.th.
